# Neuropilin 2 in osteoblasts regulates trabecular bone mass in male mice

**DOI:** 10.3389/fendo.2023.1223021

**Published:** 2023-08-01

**Authors:** Lieve Verlinden, Stefanie Doms, Iris Janssens, Mark B. Meyer, J. Wesley Pike, Geert Carmeliet, Annemieke Verstuyf

**Affiliations:** ^1^ Department of Chronic Diseases and Metabolism, Clinical and Experimental Endocrinology, KU Leuven, Leuven, Belgium; ^2^ Department of Nutritional Sciences, University of Wisconsin-Madision, Madison, WI, United States; ^3^ Department of Biochemistry, University of Wisconsin-Madision, Madison, WI, United States

**Keywords:** neuropilin 2, bone, vitamin D, osteoblast, osteoclast

## Abstract

**Introduction:**

Neuropilin 2 (NRP2) mediates the effects of class 3 semaphorins and vascular endothelial growth factor and is implicated in axonal guidance and angiogenesis. Moreover, NRP2 expression is suggested to be involved in the regulation of bone homeostasis. Indeed, osteoblasts and osteoclasts express NRP2 and male and female global *Nrp2* knockout mice have a reduced bone mass accompanied by reduced osteoblast and increased osteoclast counts.

**Methods:**

We first examined the *in vitro* effect of the calciotropic hormone 1,25-dihydroxyvitamin D_3_ [1,25(OH)_2_D_3_] on *Nrp2* transcription in osteoblasts. We next generated mice with a conditional deletion of *Nrp2* in the osteoblast cell lineage under control of the paired related homeobox 1 promoter and mice with a conditional *Nrp2* knockdown in osteoclasts under control of the Lysozyme promoter. Mice were examined under basal conditions or after treatment with either the bone anabolic vitamin D_3_ analog WY 1048 or with 1,25(OH)_2_D_3_.

**Results and discussion:**

We show that *Nrp2* expression is induced by 1,25(OH)_2_D_3_ in osteoblasts and is associated with enrichment of the vitamin D receptor in an intronic region of the Nrp2 gene. In male mice, conditional deletion of *Nrp2* in osteoblast precursors and mature osteoblasts recapitulated the bone phenotype of global *Nrp2* knockout mice, with a reduced cortical cross-sectional tissue area and lower trabecular bone content. However, female mice with reduced osteoblastic *Nrp2* expression display a reduced cross-sectional tissue area but have a normal trabecular bone mass. Treatment with the vitamin D_3_ analog WY 1048 (0.4 μg/kg/d, 14 days, ip) resulted in a similar increase in bone mass in both genotypes and genders. Deleting *Nrp2* from the osteoclast lineage did not result in a bone phenotype, even though *in vitro* osteoclastogenesis of hematopoietic cells derived from mutant mice was significantly increased. Moreover, treatment with a high dose of 1,25(OH)_2_D_3_ (0.5 μg/kg/d, 6 days, ip), to induce osteoclast-mediated bone resorption, resulted in a similar reduction in trabecular and cortical bone mass. In conclusion, osteoblastic *Nrp2* expression is suggested to regulate bone homeostasis in a sex-specific manner.

## 1 Introduction

Neuropilin 2 (NRP2), along with NRP1, are highly conserved non-tyrosine kinase transmembrane glycoprotein receptors. Neuropilins possess a large aminoterminal extracellular domain that contains different motifs through which they can bind to various signaling molecules such as class 3 semaphorin (SEMA) ligands, vascular endothelial growth factor (VEGF) A-D, fibroblast growth factor (FGF), insulin-like growth factor (IGF-1), and transforming growth factor (TGF)-β (1, 2). NRP2 is not able to convey signal transduction on its own. In the case of Sema3 signal transduction, NRP2 acts in concert with Plexin receptors, whereas it is also able to interact with other receptors such as VEGFR, IGF-IR, and TGF-βR. Consequently, various signaling complexes can be formed and this versatility permits neuropilin receptors to be involved in numerous biological processes such as axonal guidance, cardiogenesis, angiogenesis, oncogenesis, immune cell regulation, and bone homeostasis (3–6). Indeed, NRP1 mediates the osteoclast inhibitory as well as osteoblast stimulatory activities of SEMA3A as demonstrated by the osteopenic bone phenotype of transgenic mice in which the binding site for SEMA3A in the NRP1 receptor is disrupted (7). Subsequent research suggests that neuron-derived SEMA3A is responsible for the observed bone abnormalities as the bone phenotype of neuronal-specific deletion of *Sema3a* phenocopies the bone phenotype of systemic *Sema3a^-/-^* mice, whereas mice with an osteoblast-specific deletion of *Sema3a* have a normal bone mass (8). The NRP2 receptor is expressed in both osteoblasts and osteoclasts and systemic deletion of *Nrp2* results in a low bone mass phenotype, which is accompanied by a reduced osteoblast as well as an increased osteoclast number (9). Moreover, we and others have demonstrated that osteoclast progenitors that lack *Nrp2* expression display an elevated osteoclastic differentiation potential, suggesting that NRP2 negatively regulates osteoclast differentiation (9, 10). As outlined above, NRP2 can act as a co-receptor for class 3 SEMA signaling. Interestingly, many of these SEMA3 family members are direct transcriptional targets of 1,25-dihydroxyvitamin D_3_ [1,25(OH)_2_D_3_], the biologically most active form of vitamin D_3_ (11). 1,25(OH)_2_D_3_ exerts its functions through binding to the vitamin D receptor (VDR), which acts as a ligand-activated transcription factor. To regulate the transcription of primary 1,25(OH)_2_D_3_ target genes, liganded VDR interacts with the retinoid X receptor and this heterodimer binds DNA at specific vitamin D responsive elements (VDREs), preferentially DR3 type VDREs that are composed of direct repeats of two hexameric core binding sequences separated by 3 random nucleotides (12). The major role of 1,25(OH)_2_D_3_ is to maintain calcium homeostasis by stimulating intestinal absorption and renal reabsorption of calcium and by releasing calcium from skeletal stores during a negative calcium balance (12, 13). Although 1,25(OH)_2_D_3_ directly affects osteoblast differentiation, its exact signaling cascade in bone cells remains to be deciphered (14).

In the present study we investigated whether *Nrp2*, in analogy with its SEMA3 ligands, is a primary 1,25(OH)_2_D_3_-target gene in bone cells and whether it is involved in mediating the effects of 1,25(OH)_2_D_3_ on bone. In order to specifically investigate the effects of NRP2 in different bone cell types, mice with a selective depletion of *Nrp2* from either osteoblast or osteoclast progenitors were generated and examined under basal conditions or after treatment with either 1,25(OH)_2_D_3_ or with a vitamin D analog that promotes bone formation.

## 2 Materials and methods

### 2.1 Cell culture

The mouse pre-osteoblast MC3T3-E1 and mouse bone marrow-derived stromal ST2 cells (both from Riken Cell Bank, Japan) were maintained in standard growth medium [minimum essential medium alpha (αMEM) supplemented with 10% FBS, 100 units/ml penicillin and 100 µg/ml streptomycin, all from Thermo Fisher Scientific].

Primary calvarial osteoblasts were isolated from newborn mice as described previously (15). To investigate transcriptional induction of *Nrp2* by 1,25(OH)_2_D_3_, cells were incubated with vehicle (0.001% ethanol) or 1,25(OH)_2_D_3_ (10^-8^ M) in the presence or absence of actinomycin D [30 min pretreatment with actinomycin D (5 µg/ml) and 6 h treatment with 1,25(OH)_2_D_3_ (10^-8^ M) and actinomycin D] or cycloheximide (100 ng/ml, 12 h) (Merck). To assess mineralization, primary osteoblasts were cultured until confluency in standard growth medium and then switched to osteogenic medium [α-MEM with 15% FBS, 50 µg/ml ascorbic acid (Merck), 10 mM β-glycerophosphate (Merck), 10^-8^M dexamethasone (Merck), 100 units/ml penicillin and 100 µg/ml streptomycin]. After 14 days, cultures were stained with alizarin red to evaluate mineralization.

Osteoclast differentiation was examined in *in vitro* cultures of hematopoietic bone marrow cells incubated with exogenous macrophage colony-stimulating factor (M-CSF, R&D Systems) and receptor activator of nuclear factor κB ligand (RANKL, Peprotech). Bone marrow cells, from hind- and forelimbs of 8- to 10-week-old mice, were centrifuged on a Ficoll-Pague gradient (Stem Cell Technologies) to isolate mononuclear cells, which were plated overnight in αMEM containing M-CSF (10 ng/ml), penicillin (100 units/ml), and streptomycin (100 µg/ml). The non-adherent cells were harvested at day 1 and plated at a density of 120,000 cells per cm^2^ in αMEM containing M-CSF (20 ng/ml), RANKL (100 ng/ml), penicillin (100 units/ml), and streptomycin (100 µg/ml). Medium was replaced after 3 days and tartrate-resistant acid phosphatase (TRAP) staining was performed at day 6 to assess osteoclastogenesis as described previously (9). At least two technical replicates were included for each independent measurement.

For cocultures of primary osteoblasts and osteoclasts, mouse bone marrow cells were isolated from femurs and tibias of 7- to 9-week-old mice by flushing the bones with standard growth medium, passing the cells through a 70 μm cell strainer and pelleting them by centrifugation at 1330 rpm for 5 min. Osteoclasts were generated by coculturing the freshly isolated bone marrow cells (200,000 cells/cm^2^) with primary osteoblasts (20,000 cells/cm^2^) in the presence of 10^-9^ M 1,25(OH)_2_D_3_ and prostaglandin E_2_ (10^-6^ M). Culture medium was replaced every 2 days and after 6 days the cultures were stained for TRAP to visualize and count the osteoclasts or RNA was isolated. At least two technical replicates were performed for each independent measurement.

### 2.2 Chromation immunoprecipitation and ChIP-seq analysis

ChIP-seq analyses were previously performed in MC3T3-E1 cells (GSE51515 and GSE41955) (16, 17) and performed as described previously (11).

### 2.3 Transient transfection experiments

A genomic DNA fragment (mm9) of *Nrp2* (Chr1: 62,768,609-62,768,872) was cloned in the pGL3-Basic luciferase reporter vector (Promega). Four nucleotides of the DR3-type VDRE in this genomic fragment were mutated with the QuickChange II Site-Directed mutagenesis kit (Stratagene) and the resulting fragment was cloned in the pGL3-Basic luciferase reporter vector. Exponentially growing MC3T3-E1 cells were transfected with Fugene 6 transfection reagent (Roche) with one of these reporter vectors (200 ng) together with the pcDNA3.1(-)/myc-His/lacZ vector (20 ng, Thermo Fischer Scientific) to assess transfection efficiency. After transfection, cells were cultured overnight and stimulated the following day with vehicle or 1,25(OH)_2_D_3_ (10^-8^ M). After 24 h, luciferase activity was assessed and normalized to β-galactosidase activity. At least 2 technical replicates were performed for each independent measurement.

### 2.4 Adhesion and migration assays

Primary calvarial osteoblasts were cultured for 48 h in standard growth medium. Prior to the experiments, medium was switched for 4 h to αMEM containing 0.1% BSA.

For adhesion experiments, 96-well dishes were coated overnight with 10 µg/ml fibronectin, washed with αMEM containing 0.1% BSA, and blocked for 2 h with αMEM containing 0.5% BSA. Primary osteoblasts (30,000 cells/cm^2^) were seeded on fibronection-coated wells in αMEM containing 0.1% BSA, and allowed to adhere for 30 min at 37°C. Afterwards, the 96-well dish was vigorously shaken for 10 sec on a rocking platform at 2000 rpm, and wells were washed with αMEM containing 0.1% BSA. Adherent cells were fixed for 10 min with 4% paraformaldehyde (PFA), washed with PBS, and stained for 10 min with crystal violet (5 mg/ml in 2% ethanol). Wells were washed with distilled water, air dried, incubated with 2% sodium dodecyl sulphate (SDS, Thermo Fisher Scientific) at room temperature (RT) for 30 min, and absorbance was measured at 590 nm.

For migration experiments, the underside of polycarbonate membranes of Boyden chambers (8 µm pores, Corning) was coated overnight with 2.5 µg/ml fibronectin. Afterwards, primary osteoblasts (30,000 cells/well) were seeded in the upper wells of the coated Boyden chambers in αMEM containing 0.1% BSA. Cells were allowed to migrate for 4 h to the lower compartment of the Boyden chambers, which were filled with standard growth medium. Thereafter, cells at the upper side of the carbonate filters were removed and cells at the lower side were fixed for 10 min with 4% PFA, washed with PBS, and stained for 10 min with crystal violet (5 mg/ml in 2% ethanol). Carbonate filters were washed with distilled water, air dried, incubated with 2% SDS at RT for 30 min, and absorbance measured at 590 nm. At least two technical replicates were performed for each independent measurement.

### 2.5 Transgenic mice

*Nrp2* heterozygous and *Nrp2^lox/lox^* mice on C57Bl/6 background were kindly provided by D. Ginty (The Johns Hopkins University School of Medicine, Baltimore, MD) and were maintained in a conventional animal facility (18). *B6.Cg-Tg(Prrx1-cre)1Cjt/J* and *B6.129P2-Lyz2^tm1(cre)Ifo^/J* mice were obtained from the Jackson Laboratory. Mice with a conditional deletion of *Nrp2* in osteoblast precursors and mature osteoblasts were generated by crossing paired related homeobox 1 (*Prrx1)*-*Cre* mice with *Nrp2^lox/lox^* mice, yielding osteoblast-specific *Nrp2* knockdown mice (*Nrp2^Ob-^* mice). Mice with a conditional deletion of *Nrp2* in osteoclast precursors and mature osteoclasts were generated by crossing Lysozyme 2 (*Lyz2)*-*Cre* mice with *Nrp2^lox/lox^* mice, yielding osteoclast-specific *Nrp2* knockdown mice (*Nrp2^Oc-^* mice). Genotyping was performed by polymerase chain reaction (PCR) on genomic DNA from toe clips obtained from preweanling mice. All animal experiments were approved by the ethical committee of the KU Leuven (P090/2019).

### 2.6 Serum biochemistry

Serum N-terminal propeptide of type I procollagen (PINP) levels and degradation products of C-terminal telopeptides of type II collagen (CTX) were determined by ELISA according to the manufacturer’s recommendations (ImmunoDiagnostic Systems).

### 2.7 RNA extraction, cDNA synthesis, and qPCR

Total RNA was isolated, cDNA prepared with oligo d(T)16 primers (Thermo Fisher Scientific), and quantitative real-time PCR (qPCR) reactions performed with 1/10 diluted cDNA templates with the Fast SYBR Green Master Mix (Thermo Fisher Scientific) or the TaqMan Fast Universal Master Mix (Thermo Fisher Scientific) as described previously (19). Relative gene expression was calculated using the 2^-ΔCt^ method and the reference genes *β-actin* and/or *Hprt* were used to normalize gene expression. Primers and probes were obtained from Integrated DNA Technologies and sequences are available upon request.

### 2.8 Western blotting

Total protein was isolated from primary osteoblast and osteoclast cultures. Protein isolation and Western blotting was performed as described in Verlinden et al. (9) Briefly, total protein extracts were isolated and 20 µg of protein was separated by SDS-polyacrylamide gel electrophoresis (PAGE) using 4-12% polyacrylamide gels (Thermo Fisher Scientific) and transferred to a nitrocellulose membrane (GE Health care). Membrane blocking was done in TBS (10 mM Tris-HCl, pH 7.6; 150 mM NaCl) with 5% non-fat dry milk and 0.1% Tween 20 (Merck). The rabbit monoclonal (D39A5) anti-mouse NRP2 antibody (CST) was diluted 1/500, while the mouse anti-actin (Merck) was diluted 1/5000. Incubation with a secondary antibody (Dako) was performed for 1 h at RT, and enhanced chemoluminescence (Perkin Elmer) was used to visualize protein bands.

### 2.9 Microcomputed tomography

The high resolution Skyscan 1272 system (Bruker) was used for µCT analysis with the following parameters: 60 kV as source voltage, 83 μA as source current, 0.5 mm aluminum filter, 5 µm pixel size, 0.4° rotation step. Data reconstruction was performed with NRecon (Bruker) and data analysis with CTAn (Bruker). Trabecular bone parameters were defined in a volume of interest (VOI) between 1 and 2.5 mm below the growth plate, whereas cortical bone parameters were quantified in a VOI between 2.5 to 3 mm below the growth plate. Analysis was performed according to the guidelines of the American Society for Bone and Mineral Research (20). 3D models were constructed with CTvox software (Bruker).

### 2.10 Histological analysis

PFA-fixed bones were decalcified in 0.5 M ethylenediamine tetraacetic acid (EDTA) in PBS for 2 weeks at 4°C, dehydrated in graded ethanol concentrations, and embedded in paraffin. Paraffin sections (4 µm) were cut, dewaxed in xylene, and rehydrated before staining. For general morphological analysis, paraffin sections were stained in hematoxylin (Prosan) and eosin (H&E; 0.6% eosin Y, 1% phloxine B, 2% orange G, Merck) for 30 sec and 2 min, respectively. Paraffin sections were stained for TRAP activity and counterstained with Light Green SF Yellowish (Merck) to quantify osteoclasts (15). NRP2 immunostaining was performed as described previously by Verlinden et al. (9)

### 2.11 Bone histomorphometry

Bones were fixed for 24 h in 2% PFA, rinsed and stored in PBS. After decalcification, bones were embedded in paraffin and 4 µm thick sections were used for stainings. Osteoclasts and osteoblasts covering the trabecular surfaces were measured on 3 TRAP- and H&E- stained sections, respectively, and expressed relative to the total bone surface (21). In each section, three consecutive fields (0.8 mm width en 0.4 mm height) were measured along the vertical axis of the central metaphysis, starting 0.2 mm from the distal end of the growth plate as described previously by Verlinden et al. (9).

### 2.12 Statistical analysis

Results from *in vitro* experiments are expressed as mean and SEM, whereas data from *in vivo* measurements are expressed as mean and SD. To assess differences between two experimental groups, two-tailed Student’s t-tests were performed. To determine the effect of selective *Nrp2* deletion in male and female mice, two-way ANOVA was performed with genotype and sex as two independent variables, followed by Sidak’s multiple comparisons test. To evaluate the effect of treatment with 1,25(OH)_2_D_3_ or the vitamin D analog, MV2, in osteoblast- or osteoclast-specific *Nrp2^-/-^* mice, we performed two-way ANOVA analysis, with genotype and treatment as independent variables, followed by Sidak’s multiple comparisons test.

## 3 Results

### 3.1 *Nrp2* is a primary vitamin D target gene

**Figure 1:**
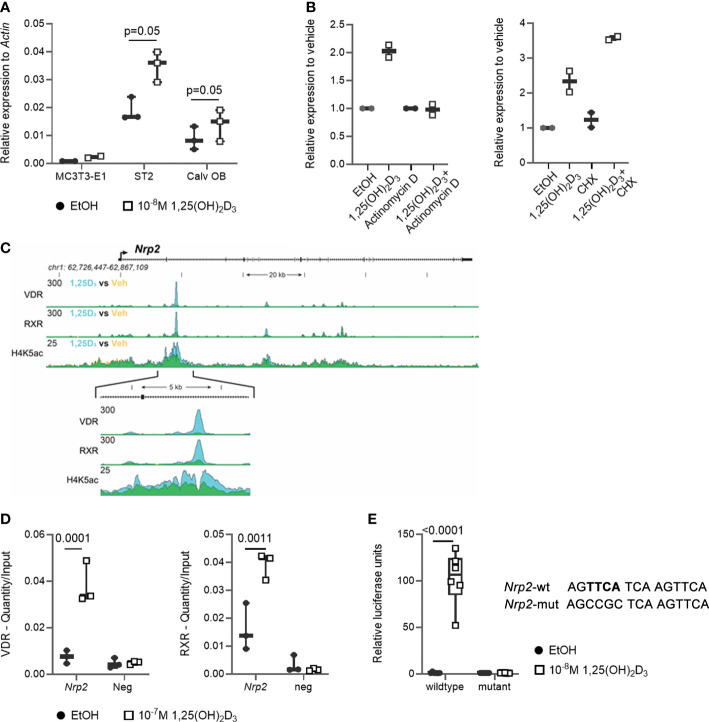
*Nrp2* is a direct transcriptional target of 1,25(OH)_2_D_3_. **(A)** *Nrp2* transcript levels are induced in MC3T3-E1 and ST2 osteoblast cell lines and in primary calvarial osteoblasts after treatment with 1,25(OH)_2_D_3_ (24 h, 10^-8^ M) (n=2-3). **(B)** *Nrp2* transcript levels in MC3T3-E1 cells stimulated with 1,25(OH)_2_D_3_ (10^-8^ M) and treated with actinomycin D (5 µg/ml, 6 h) or cycloheximide (CHX) (100 ng/ml, 12 h) (n=2). **(C)** Overlaid triplicate and averaged ChIP-Seq tracks for VDR, RXR, and H4K5 acetylation (H4K5ac) where ethanol vehicle (Veh) are shown in yellow, 1,25(OH)_2_D_3_ treatment shown in blue, and overlapping data appear as green. *Nrp2* genomic region displayed is chr1:62,726,447-62,867,109 with exons depicted as boxes, introns by arrows. Region of interest is highlighted in the inset below (chr1:62,765,185-62,770,248). Maximum height of tag sequence density for each data track indicated on the Y-axis (normalized to input and 10^7^ tags). **(D)** ChIP-qPCR results confirmed the 1,25(OH)_2_D_3_-inducible association of VDR and RXR in response to treatment at the studied VDR binding site within the *Nrp2* gene. ChIP was performed with chromatin samples from MC3T3-E1 cells incubated with 1,25(OH)_2_D_3_ (10^-7^ M) or vehicle for 3 h (n=3). **(E)** A DNA fragment, containing either the wildtype or a mutated, non-functional VDR binding site, was cloned and inserted into the pGL3-basic vector. Luciferase reporter gene assays were performed in exponentially growing MC3T3-E1 cells to evaluate the functionality of the identified VDR binding sites (10^-8^ M 1,25(OH)_2_D_3_, 24 h). Luciferase activity was normalized to that of empty pGL3-basic reporter vector (n=3). All results are expressed as mean and SEM. Two-tailed Student’s t-tests were performed to determine significant differences between 1,25(OH)_2_D_3_-treated and vehicle-treated cells.

We previously demonstrated that transcript levels of the majority of SEMA3 family members are directly transcriptionally regulated by 1,25(OH)_2_D_3_ (11). Therefore, we investigated the expression of their co-receptor *Nrp2* in osteoblasts. *Nrp2* transcript levels were present in the mouse (pre-) osteoblast MC3T3-E1 and ST2 cell lines as well as in primary mouse calvarial osteoblasts and its transcript levels were induced approximately 2-fold after a 24 h incubation with 1,25(OH)_2_D_3_ (10^-8^ M) (**Figure 1A**). Interestingly, in the presence of the transcriptional inhibitor actinomycin D, incubation with 1,25(OH)_2_D_3_ did not result in elevated *Nrp2* transcript levels, whereas transcript levels were induced upon co-incubation of 1,25(OH)_2_D_3_ with the protein synthesis inhibitor cycloheximide (CHX) (**Figure 1B**). ChIP-seq analysis demonstrated direct VDR and RXR binding as well as elevated H4 acetylation in the second intron of the *Nrp2* gene (**Figure 1C**). Further ChIP-qPCR analysis confirmed 1,25(OH)_2_D_3_-inducible association of VDR and RXR with this genomic region (**Figure 1D**). *In silico* analysis revealed the presence of a DR3 type VDRE in this VDR- and RXR-bound genomic region under the peak maxima aligning where the H4K5 acetylation was vacated. The functionality of this VDRE was demonstrated in transient transfection experiments. Indeed, transcription of a luciferase reporter vector driven by the VDRE-containing genomic region was significantly elevated after treatment with 1,25(OH)_2_D_3_, and mutation of the putative VDRE completely abrogated the 1,25(OH)_2_D_3_-mediated transcriptional induction of *Nrp2* (**Figure 1E**).

In conclusion, these data demonstrate that osteoblasts express NRP2 and that its transcription is directly increased by 1,25(OH)_2_D_3_ through elevated VDR/RXR binding to a newly identified VDRE.

### 3.2 Osteoblast-specific deletion of *Nrp2* reduces trabecular bone mass only in male mice, but decreases bone diameter in both male and female mice

**Figure 2:**
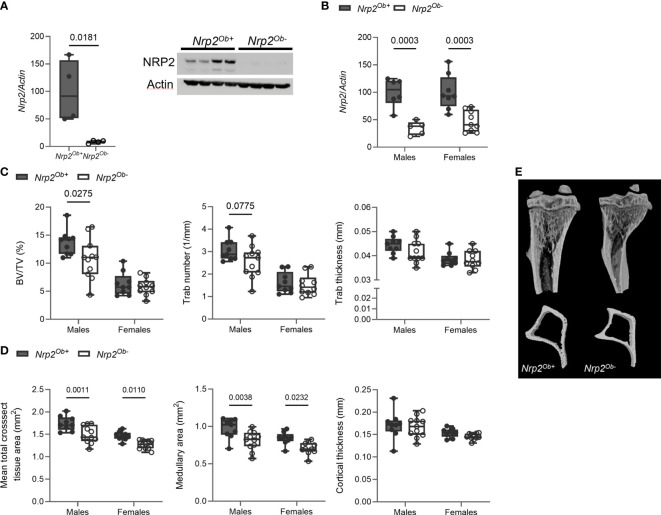
Osteoblast-specific deletion of *Nrp2* affects bone differently in male and female mice. **(A)** NRP2 transcript and protein levels were significantly lower in primary calvarial osteoblasts derived from *Nrp2^Ob-^* mice compared to osteoblasts from wildtype littermates (n=4). Data are expressed as mean and SEM. Two-tailed Student’s t-tests were performed to detect significant differences **(B)** Reduced *Nrp2* transcript levels in whole bone homogenates from 10-week-old male and female *Nrp2^Ob-^* mice (n=5-9). **(C, D)** µCT analysis and evaluation of trabecular **(C)** and cortical bone parameters **(D)** in tibia of 10-week-old male and female *Nrp2^Ob-^* mice and their wildtype littermates (n=9-11). **(E)** Representative 3D models of tibial epiphyses and metaphyses (upper panel) and cross-sections of the tibial mid diaphysis from 10-week-old male *Nrp2^Ob-^* mice and their wildtype littermates. Data from panels B-E are expressed as mean and SD. Two-way ANOVA analysis, with genotype and sex as independent variables, followed by Sidak’s multiple comparisons test, was performed to evaluate significant differences between genotypes for male and female mice.

Our previous findings indicated that NRP2 expression is involved in normal bone homeostasis both in male (9) and female mice (**Supplementary Figure 1**). To investigate whether the effects of NRP2 were mediated systemically or rather by NRP2 expression specifically in bone cells, we deleted *Nrp2* in osteoblasts and its progenitors (*Nrp2^Ob-^* mice) by crossing *Prrx1*-Cre mice with *Nrp2^lox/lox^* mice. NRP2 expression was significantly lower in primary calvarial osteoblasts isolated from *Nrp2^Ob-^* than from *Nrp2^Ob+^* mice, confirming an efficient deletion of NRP2 in osteoblasts (**Figure 2A**). In addition, *Nrp2* transcript levels were significantly lower in whole bone RNA extracts from *Nrp2^Ob-^* mice, compared to *Nrp2^Ob+^* mice in both male and female mice (**Figure 2B**). Because the *Prrx1* promoter targets osteochondroprogenitors, we also evaluated NRP2 expression in chondrocytes and adipocytes. Immunohistochemical analysis did not demonstrate NRP2 protein expression in chondrocytes (9). qPCR analysis of white adipose tissue (WAT) indicated that *Nrp2* is expressed at low levels in adipocytes, however, no difference in *Nrp2* expression was observed in WAT derived from *Nrp2^Ob+^* and *Nrp2^Ob-^* mice (data not shown).

Tibia length was not different between 8-week-old *Nrp2^Ob-^* mice and their wildtype littermates (data not shown). Interestingly, µCT analysis of the tibia of 8-week-old mice demonstrated a significant reduction of trabecular bone mass (BV/TV) in male, but not in female mice (**Figure 2C**). The reduced trabecular bone mass in male mice mainly resulted from a reduction in trabecular number and a tendency towards a decreased trabecular thickness (**Figures 2C, E**). Remarkably, tibias of female and, especially, male mice at 8 weeks of age were characterized by a reduction in mean total cross-sectional tissue area and medullary area, whereas cortical thickness (**Figures 2D, E**) and cortical porosity (data not shown) were not altered. This decrease in bone diameter progressed with older age as illustrated by µCT analysis and 3D reconstructions of tibias of 26-week-old females, while no significant changes in trabecular bone parameters were observed at this age (**Supplementary Figure 2A**).

**Figure 3:**
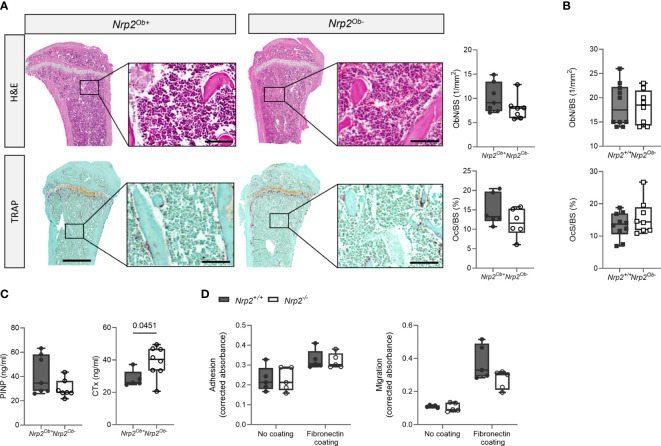
Osteoblast and osteoclast activity in *Nrp2^Ob-^* mice. **(A)** Representative pictures and histomorphometric analysis of osteoblasts (upper panels, n=6-7) and osteoclasts (lower panels, n=5-6) in tibia of 10-week-old male *Nrp2^Ob-^* mice and wildtype littermates (scale bars are 500 µm in overview pictures and 100 µm in picture details). **(B)** Quantification of osteoblast number and osteoclast surface in tibia of 8-week-old female *Nrp2^Ob-^* mice and wildtype littermates (n=8-10). **(C)** Serum P1NP and CTx levels in 10-week-old male *Nrp2^Ob-^* mice and wildtype littermates (n=5-7). **(D)** *In vitro* evaluation of adhesive and migratory capacity of primary *Nrp2^-/-^* and *Nrp2^+/+^* calvarial osteoblasts (n=4-5). All *in vivo* data are expressed as mean and SD, whereas *in vitro* data are expressed as mean and SEM. Two-tailed Student’s t-tests were performed to detect significant differences.

Despite the reduced trabecular bone mass in male *Nrp2^Ob-^* mice, bone histomorphometry revealed no differences in osteoblast number or in osteoclast surface between *Nrp2^Ob-^* mice and their wildtype littermates (**Figure 3A**). Also in female mice, histomorphometric analysis did not reveal any effect of *Nrp2* deletion on osteoblast or osteoclast numbers (**Figure 3B**). Serum P1NP levels, a marker for osteoblast activity, tended to be slightly lower in male *Nrp2^Ob-^* mice, however, this difference did not reach significance (**Figure 3C**, left panel), whereas serum levels of CTx, a marker for osteoclast activity, were significantly increased in *Nrp2^Ob-^* mice (**Figure 3C**, right panel). Transcript analysis of whole bone homogenates from *Nrp2^Ob-^* mice and their wildtype littermates did not show any differences in expression of osteoblast (osteocalcin, osteopontin) or osteoclast (cathepsin K) markers in male or female mice (**Supplementary Figures 2B, C**). To investigate whether *Nrp2* deletion in calvarial osteoblasts affected their intrinsic differentiation capacity, we performed *in vitro* mineralization studies. However, alizarin red staining and qPCR analysis did not show any differences in mineralization efficacy of osteoblasts derived from *Nrp2^Ob-^* mice or from their wildtype littermates (**Supplementary Figure 3**). Finally, as NRP2 can act as a coreceptor for different semaphorin ligands, which are involved in the regulation of cell adhesion and migration, we evaluated whether osteoblastic *Nrp2* deletion affected their adhesive and migratory capacity. As can be observed in **Figure 3D**, fibronectin coating enhanced cell adhesion and induced cell migration, however, to the same extent for *Nrp2^+/+^* and *Nrp2^-/-^* osteoblasts. Similar results were obtained for vitronectin coating (data not shown).

In conclusion, these data indicate that there is a sex-specific role for osteoblastic *Nrp2* expression in the control of trabecular bone mass. However, both *in vitro* and *in vivo* analyses failed to demonstrate an effect of osteoblastic *Nrp2* expression on osteoblast adhesion, migration, or mineralization.

### 3.3 *Nrp2*-specific deletion in osteoclasts does not affect bone homeostasis

**Figure 4:**
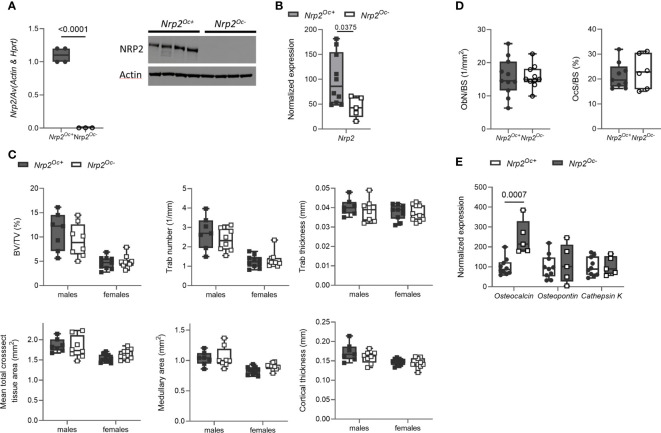
Osteoclast-specific deletion of *Nrp2* does not affect bone mass. **(A)** NRP2 transcript and protein levels were significantly lower in primary osteoclast cultures derived from hematopoietic cells that were isolated from *Nrp2^Oc-^* or *Nrp2^Oc+^* mice and *in vitro* stimulated with M-CSF and RANKL (n=3-4). Data are expressed as mean and SEM. Two-tailed Student’s t-tests were performed to detect significant differences. **(B)** Reduced *Nrp2* transcript levels in whole bone homogenates from 10-week-old female *Nrp2^Oc-^* mice compared to wildtype littermates (n=5-10). Data are expressed as mean and SS. Two-tailed Student’s t-tests were performed to detect significant differences. **(C)** µCT analysis and evaluation of trabecular and cortical bone parameters in tibia of 10-week-old male and -female *Nrp2^Oc-^* mice and their wildtype littermates (n=7-9). Data are expressed as mean and SD. Two-way ANOVA analysis, with genotype and sex as independent variables, followed by Sidak’s multiple comparisons test, was performed to evaluate significant differences between genotypes for male and female mice. **(D)** Histomorphometric analysis of osteoblast numbers (left panel) and osteoclast surface (right panel) in tibia of 10-week-old female *Nrp2^Oc-^* mice and their wildtype littermates (n=6-11). Data are expressed as mean and SD. Two-tailed Student’s t-tests were performed to detect significant differences. **(E)** qPCR analysis of the osteoblast markers *osteocalcin* and *osteopontin* and the osteoclast marker *cathepsin K* in whole bone homogenates of 10-week-old female *Nrp2^Oc-^* mice and their wildtype littermates. Data are expressed as mean and SD. Two-tailed Student’s t-tests were performed to detect significant differences.

**Figure 5:**
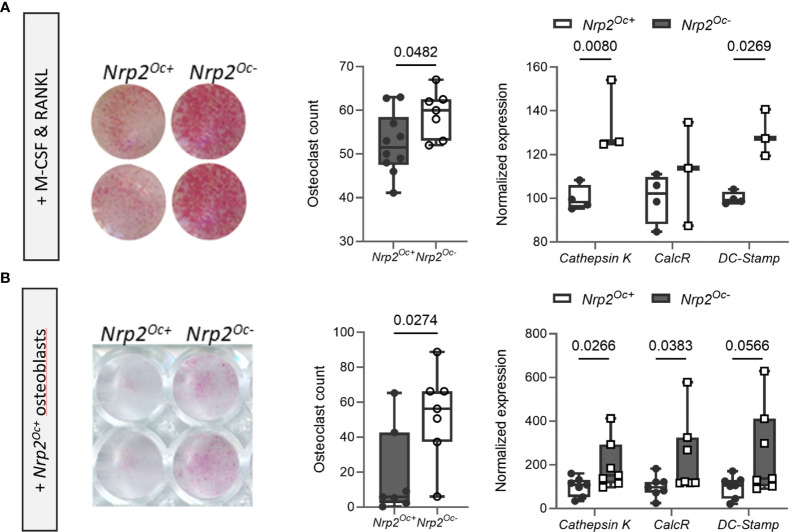
*In vitro* osteoclastogenesis is enhanced when hematopoietic cells are derived from *Nrp2^Oc-^* mice. **(A)** Hematopoietic cells, derived from *Nrp2^Oc-^* mice or their wildtype littermates, were cultured in presence of M-CSF and RANKL. Osteoclasts were quantified on TRAP stainings (n=7-10) and qPCR analysis was performed to evaluate expression of the osteoclast-specific genes *cathepsin K, calcitonin receptor* and *DC-stamp* (n=3-4). **(B)** Hematopoietic cells, derived from *Nrp2^Oc-^* mice or their wildtype littermates, were co-cultured with wildtype osteoblasts and stimulated with PGE_2_ and 1,25(OH)_2_D_3_. Osteoclasts were quantified on TRAP stainings (n=7) and qPCR analysis was performed to evaluate expression of the osteoclast-specific genes *cathepsin K*, *calcitonin receptor* and *DC-stamp* (n=7). Data are expressed as mean and SEM. Two-tailed Student’s t-tests were performed to detect significant differences.

Previous research from our group demonstrated that systemic *Nrp2* deficiency is associated with an increased osteoclast number in bone and that hematopoietic cells derived from *Nrp2^-/-^* mice differentiate more efficiently into osteoclasts (9). We therefore examined whether *Nrp2* deletion specifically in osteoclasts and its precursors (*Nrp2^Oc-^* mice) affected osteoclast formation and bone homeostasis by crossing *Nrp2^lox/lox^* mice with *LysM-Cre* mice. Osteoclasts, generated by culturing hematopoietic cells from *Nrp2^Oc-^* mice in the presence of M-CSF and RANKL, did not express NRP2 transcript or protein, confirming an efficient NRP2 deletion in osteoclasts (**Figure 4A**). In addition, *Nrp2* transcript levels were significantly reduced in RNA extracts of whole bone homogenates from *Nrp2^Oc-^* mice (**Figure 4B**). However, this substantial reduction in osteoclastic *Nrp2* expression did not affect bone homeostasis in male nor female mice as µCT analysis did not reveal any differences in trabecular or cortical bone mass, nor in cross-sectional bone area between 8-week-old *Nrp2^Oc+^* and *Nrp2^Oc-^* mice (**Figure 4C**). Also at 16 weeks of age, no differences in bone mass between female *Nrp2^Oc+^* and *Nrp2^Oc-^* mice were observed (**Supplementary Figure 4**). Concordantly, bone histomorphometry did not show differences in osteoblast number or osteoclast surface between *Nrp2^Oc+^* and *Nrp2^Oc-^* mice in females (**Figure 4D**). Rather surprisingly, transcript levels of osteocalcin were significantly elevated in female *Nrp2^Oc-^* mice compared to their control littermates, whereas the expression of other osteoblast (osteopontin) or osteoclast (cathepsin K) markers was not different (**Figure 4E**). In contrast to the *in vivo* findings, *in vitro* osteoclast cultures showed that osteoclast numbers and expression of osteoclast markers were increased when hematopoietic cells of *Nrp2^Oc-^* origin were used, whether cultured in the presence of M-CSF and RANKL or cocultured with calvarial osteoblasts (**Figures 5A, B**).

Collectively, these data illustrate that *Nrp2* deletion from osteoclast precursors results in an elevated *in vitro* but not *in vivo* osteoclastic differentiation potential.

### 3.4 Short-term treatment of osteoblast- and osteoclast specific *Nrp2* knockout mice with the vitamin D analog WY 1048 or 1,25(OH)_2_D_3_, respectively

**Figure 6:**
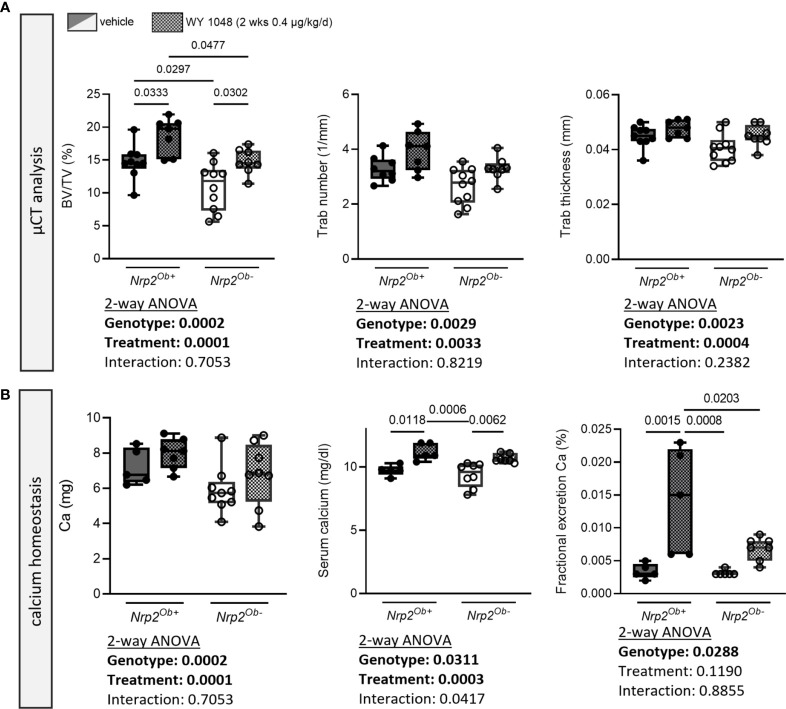
Short-term treatment with the vitamin D analog WY 1048 increases bone mass similarly in both genotypes. **(A)** µCT analysis and evaluation of trabecular bone parameters in femurs of 10-week-old male *Nrp2^Ob-^* and *Nrp2^Ob+^* mice, which were treated daily for 2 weeks with 0.4 µg/kg WY 1048 or vehicle (n=7-8). **(B)** Calcium content in bone, levels in serum, and renal fractional excretion in 10-week-old male *Nrp2^Ob-^* and *Nrp2^Ob+^* mice, treated daily with 0.4 µg/kg WY 1048 or vehicle during 2 weeks (n=5-9). All data are expressed as mean and SD. Two-way ANOVA analysis, with genotype and treatment as independent variables, followed by Sidak’s multiple comparisons test was performed to detect significant differences between *Nrp2^Ob+^* and *Nrp2^Ob-^* mice and to evaluate the effect of the treatment.

Since we have shown that *Nrp2* is a direct vitamin D target gene, we aimed to investigate whether treatment with the vitamin D analog WY 1048, which is able to prevent bone loss in an ovariectomy-induced model of osteoporosis (22), increases bone mass to the same extent in osteoblast-specific *Nrp2* knockout mice as in their wildtype littermates. Therefore, 8-week-old male *Nrp2^Ob-^* and *Nrp2^Ob+^* mice were injected daily for 2 consecutive weeks with the analog WY 1048 (0.4 µg/kg/d). In both genotypes, short-term treatment with WY 1048 resulted in a significant, but comparable increase in trabecular bone mass, which was accompanied by non-significant increases in trabecular number and thickness (**Figure 6A**). Cortical thickness was not altered upon treatment with WY 1048 (data not shown). In agreement with the elevated BV/TV after treatment with WY 1048, there was a trend towards an increased bone calcium content in WY 1048-treated mice (**Figure 6B**). In addition, serum calcium levels as well as renal fractional excretion of calcium were similarly elevated in *Nrp2^Ob-^* and *Nrp2^Ob+^* mice after treatment with WY 1048 (**Figure 6B**), illustrating that short-term treatment with the vitamin D analog WY 1048 has similar bone anabolic effects in *Nrp2^Ob-^* and *Nrp2^Ob+^* mice, which are accompanied by a limited increase in serum and urinary calcium levels.

**Figure 7:**
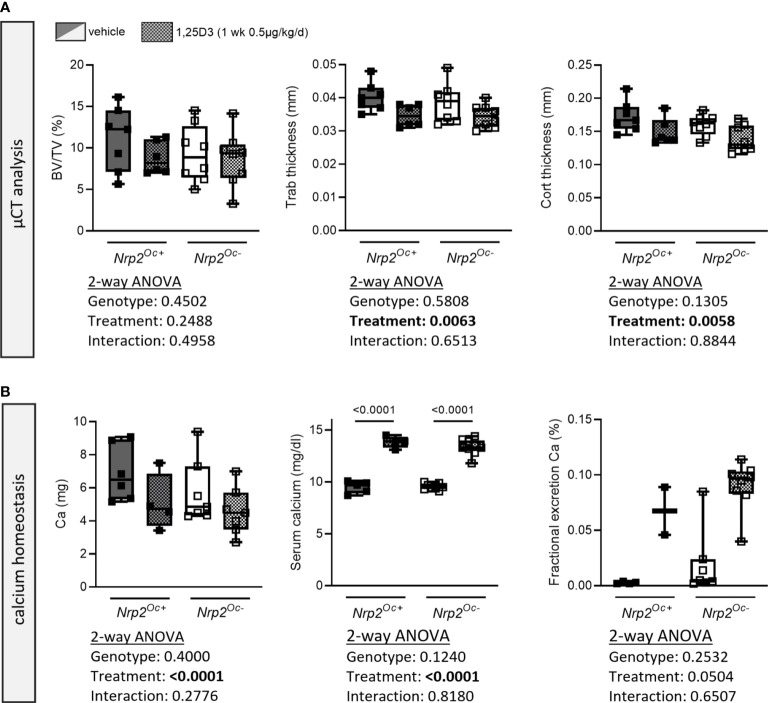
Short-term treatment with 1,25(OH)_2_D_3_ does not affect bone differently in *Nrp2^Oc-^* mice and their wildtype littermates. **(A)** µCT analysis and evaluation of trabecular and cortical bone parameters in femurs of 10-week-old male *Nrp2^Oc-^* and *Nrp2^Oc+^* mice, which were treated daily for 1 week with 0.5 µg/kg 1,25(OH)_2_D_3_ or vehicle (n=7-8). **(B)** Calcium content in bone, serum, and urine in 10-week-old male *Nrp2^Oc-^* and *Nrp2^Oc+^* mice, daily treated with 0.5 µg/kg 1,25(OH)_2_D_3_ or vehicle during 1 week (n=5-9). All data are expressed as mean and SD. Two-way ANOVA analysis, with genotype and treatment as independent variables, followed by Sidak’s multiple comparisons test was performed to detect significant differences between *Nrp2^Oc+^* and *Nrp2^Oc-^* mice and to evaluate the effect of the treatment.

Because of the apparent discrepancy between the elevated *in vitro* osteoclast differentiation of *Nrp2^Oc–^* derived osteoclast precursors and the normal osteoclast numbers in *Nrp2^Oc-^* mice, we aimed to investigate bone homeostasis of *Nrp2^Oc-^* mice in conditions of high bone resorption, induced by daily administration of supraphysiological doses of 1,25(OH)_2_D_3_ (1 week, 0.5 µg/kg/d). As expected, trabecular bone volume and trabecular thickness as well as cortical thickness tended to decrease upon administration of 1,25(OH)_2_D_3_, however these parameters were reduced to the same extent in *Nrp2^Oc+^* and *Nrp2^Oc-^* male mice (**Figure 7A**). Also systemic calcium handling was similar in both genotypes as evidenced by the highly elevated serum calcium levels and urinary calcium excretion (**Figure 7B**). Similar results were obtained in female mice (data not shown). These data indicate that even in conditions of high bone remodeling, osteoclastic *Nrp2* deletion does not affect bone homeostasis.

## 4 Discussion

NRPs are transmembrane receptors that are expressed on a range of cell types and are able to partner with a wide variety of other transmembrane receptors and therefore they modulate numerous signaling pathways including those activated by class 3 semaphorins, VEGF, FGF, TGF-β, and IGF-1. NRP2 has two major membrane-bound isomers, NRP2a and NRP2b (23). These isoforms are identical in sequence and structure in large parts of the N-terminal domains, whereas they are different in the transmembrane and C-terminal cytoplasmic tail regions. Recent research pointed out that in human prostate cancer C4-2B and in HEK293 cells, the NRP2B variant is able to translocate to the inner nuclear membrane in response to VEGF-C (24).

Systemic *Nrp2^-/-^* mice are viable but have an aberrant neurological function and have a severe reduction of small lymphatic vessels and capillaries (18, 25–27). Moreover, male and female *Nrp2^-/-^* mice have a lower trabecular bone mass and a reduced radial bone growth, which is accompanied by a decreased osteoblast and elevated osteoclast count *in vivo* (9). This bone phenotype could be the consequence of systemic modifications such as alterations in the immune system, sex steroids, regulation of innervation, or vasculature or rather result from absent skeletal *Nrp2* expression.

Within bone, we did not detect NRP2 expression in chondrocytes of the growth plate or fracture callus, which is in agreement with earlier findings of Hecht et al. who did not detect *Nrp2* transcripts in (pre-)hypertrophic chondrocytes of E15.5 mouse limb bud sections (28). Moreover, *Nrp2^-/-^* mice have no alterations in longitudinal bone growth and do not display overt growth plate abnormalities. Therefore, we did not silence *Nrp2* expression specifically in the chondrocytes. However, *Nrp2* transcripts are detected in the periosteum and in the trabecular bone of embryonal mouse limb bud sections (28, 29), and are approximately 3-fold reduced in embryos deficient for *Runx2*, a key player in skeletal development (28). Furthermore, we demonstrated that *Nrp2* is expressed at distinct stages of osteoblast differentiation in established (pre)osteoblast cell lines as well as in primary (differentiating) osteoblasts (9, 11). Interestingly, a recent study illustrated reduced NRP2 protein levels in bone marrow stromal cells (BMSC) that lack the splicing factor y-box binding protein 1 (YBX1). YBX1 deficiency resulted in BMSC senescence and a differentiation shift from osteoblasts to adipocytes during ageing, suggesting a role for NRP2 in the regulation of osteogenic differentiation or senescence (30).

To investigate the potential role of osteoblast-specific NRP2 signaling, we silenced its expression under the control of the *Prrx1*-promoter. Much like global *Nrp2^-/-^* mice, male and female *Nrp2^Ob-^* mice displayed a cortical bone phenotype and are characterized by a reduction in mean total cross-sectional tissue area and in medullary area. However, only male *Nrp2^Ob-^* mice had a small decrease in trabecular bone mass, similar to the reduction in trabecular bone mass observed in male and female systemic *Nrp2^-/-^* mice. Treatment with the vitamin D analog WY 1048 increased trabecular bone volume to the same degree in *Nrp2^Ob-^* mice and their wildtype littermates, in both male and female mice. The mechanisms through which WY 1048 increased trabecular bone mass remain to be identified. Interestingly, treatment with the vitamin D analog eldecalcitol leads to an increase in bone mass in mice and humans through suppression of osteoclastic bone resorption. Further research indicated that eldecalcitol is not able to induce bone mass in mice with an osteoblast-specific deletion of the *Vdr*, suggesting that suppression of osteoclast activity is mediated by osteoblast-lineage derived VDR (14). In addition, the mechanisms underlying the bone phenotype of *Nrp2^Ob-^* mice are not yet elucidated. Unfortunately, bone histomorphometric evaluation as well as analysis of osteoblast and osteoclast markers failed to detect any alterations in bone formation or resorption *in vivo*. Moreover, *in vitro* assays did not show any overt aberrancies in differentiation, adhesion, and migration capacities of osteoblasts lacking *Nrp2* expression. One of the major regulators of radial bone growth is IGF-1 and, interestingly, circulating serum IGF-1 levels were slightly decreased in systemic *Nrp2^-/-^* mice (data not shown). Moreover, NRP2, in concert with VEGF, regulates IGF-1R expression and signaling in prostate cancer cells (31). However, transcript levels of *Igf1*, *Igf1r*, *Ifgbp3*, and the more abundant *Igfbp5* were not different between *Nrp2^Ob-^* mice and their wildtype littermates (data not shown). Remarkably, the trabecular bone phenotype of *Nrp2^-/-^* mice appears to be recapitulated only in male *Nrp2^Ob-^* mice, possibly suggesting a link with androgen receptor (AR) signaling. A recent study revealed that the NRP2B transcript variant reduces the transcriptional activity of the AR in advanced prostate cancer by altering AR binding to the regulatory regions of AR target genes (24). In addition, overexpression of the NRP2A isoform in human prostate cancer cells reduces AR protein levels, represses the activity of an AR-responsive reporter construct, and inhibits the expression of AR-target genes (32). However, within bone AR-mediated signaling pathways are mainly involved in the regulation of radial bone growth as removal of the AR in male mice results in smaller and thinner bones (33, 34), whereas the reduction in bone diameter was observed both in male and female *Nrp2^Ob-^* mice.

In addition to osteoblasts and osteoclasts, other cell types in bone may express the NRP2 receptor and NRP2 expression in these cells may be involved in bone homeostasis. Osteocytes, for example, account for approximately 95% of adult bone cells and are involved in the regulation of bone remodeling, in sensing and response to mechanical stimuli, in the control of phosphate homeostasis, and in the response to hormonal signals (35). Interestingly, histological analysis revealed NRP2 protein in the osteocytes, embedded in the cortical bone (9). In addition, Zimmerman et al. also illustrated *Nrp2* transcript levels in osteocytes and showed that these levels are two-fold upregulated in two different mouse models of osteogenesis imperfecta (35). Therefore, in view of the orchestrating effects of osteocytes in bone remodeling, it would be interesting to further explore the role of osteocytic NRP2 signaling in bone homeostasis.

Recent research demonstrated the presence of lymphatic vessels in murine and human bone and revealed that VEGF-C/VEGFR-3 signaling drives lymphangiogenesis in bone (36). Moreover, genotoxic stress, provoked by whole bone irradiation or chemotherapy, promotes the expansion of lymphatic vessels and supports the generation of bone and of the hematopoietic stem cells within bone. Previous studies illustrated that NRP2 can also bind VEGF-C and that the activation of the VEGF-C/NRP2 signaling axis is closely related to lymphangiogenesis (37). Therefore, it might be interesting to examine whether lymphatic vessels within the bone express the NRP2 receptor and whether a potential crosstalk between bone lymphatic vessels and osteoblasts affects bone homeostasis.

Within bone, NRP2 is not only expressed in osteoblast lineage cells, but also in osteoclasts. Indeed, we and others demonstrated that NRP2 transcript and protein levels are increased during osteoclast differentiation (9, 10). Moreover, *Nrp2* deletion in osteoclasts (or its precursors) is accompanied by elevated osteoclastogenesis when cultured in presence of M-CSF and RANKL (9, 10) or when incubated with conditioned medium of metastatic prostate cancer cells (10). Mechanistically, *Nrp2* deletion in osteoclasts causes the calcium channels to release calcium from the endoplasmic reticulum stores resulting in elevated intracellular calcium concentrations, which in turn trigger hyperactivation of nuclear factor of activated T cells (NFATc1), a key transcription factor governing osteoclastogenesis (10). Surprisingly, however, our results indicated that *Nrp2* silencing in osteoclasts did not result in an *in vivo* bone phenotype. Moreover, others have shown that specific *Nrp2* abrogation in osteoclasts decreases the *in vivo* capacity of prostate cancer cells to metastasize to the bone (10). These discrepancies between *in vitro* and *in vivo* findings may have various reasons. As outlined above, *Nrp2* deletion from osteoclasts altered intracellular calcium concentrations and NFATc1 signaling in *in vitro* cultures (10). However, a myriad of complex signaling pathways is also able to modulate intracellular calcium concentrations (38) and we hypothesize more regulators of this pathway to be present in an *in vivo* setting. Moreover, in our *in vitro* studies we applied 2D culture models on plastic substrates, whether or not in the presence of osteoblasts, where physiological cell-cell and cell-matrix interactions are largely lacking. Indeed, it is known that interactions between bone cells and the extracellular matrix are essential for their polarization and differentiation and may result in aberrant autocrine and paracrine signaling activities affecting the bone cell phenotype (39). Together these findings indicate that *in vitro* cell culture systems cannot recapitulate the complex communication between different cells in the bone, which collectively regulate bone homeostasis.

Since *Nrp2* is expressed in various tissues and is involved in multiple cellular processes, it is not surprising that its transcription is controlled, either in a positive or negative manner, by a large number of transcriptional regulators, including different members of the nuclear receptor superfamily. Indeed, *Nrp2* expression is transcriptionally repressed by the AR (32). In addition, nuclear receptor subfamily 2, group F, member 2 (Nr2f2), a member of the steroid thyroid hormone superfamily of nuclear receptors, regulates *Nrp2* expression in murine lymphatic vessel development (40) and in the developing mouse brain (41). Our data demonstrate that *Nrp2* expression was rapidly induced by 1,25(OH)_2_D_3_ not only in osteoblast cell lines but also in primary osteoblasts, suggesting that *Nrp2* is a conserved 1,25(OH)_2_D_3_ target over different osteoblastic cell types. ChIP seq data in MC3T3-E1 cells revealed the presence of strongly inducible VDR/RXR heterodimers, accompanied by an accessible chromatin structure at the second intron of the *Nrp2* gene, pointing to the presence of a VDR binding site in the *Nrp2* gene. Given the robust nature of the ligand-induced VDR/RXR binding along with the sharp absence of H4K5 acetylation, a DR3-type VDRE was easily identified by in silico interrogation of the genomic DNA and that VDRE is able to transactivate a reporter gene in response to 1,25(OH)_2_D_3_. All together these data provide strong evidence that 1,25(OH)_2_D_3_ directly controls *Nrp2* expression in osteoblastic cells. However, the anabolic effects of the synthetic vitamin D analog WY 1048 or the bone resorbing effects of supraphysiological doses of 1,25(OH)_2_D_3_ were not dependent on the expression of *Nrp2* in osteoblastic or osteoclastic cells, respectively.

In conclusion, this study demonstrated that *Nrp2* expression in osteoblastic cells affects bone homeostasis in a sex-specific manner as male *Nrp2^Ob-^* mice have a reduced trabecular bone mass as well as a reduced cross-sectional tissue area whereas female mice only have a decreased cross-sectional tissue area. In addition, osteoclast-specific *Nrp2* deletion does not result in an altered bone homeostasis, despite an increased *in vitro* ability of *Nrp2* deficient hematopoietic cells to differentiate towards osteoclasts.

## Data availability statement

The original contributions presented in the study are included in the article/**Supplementary Material**. Further inquiries can be directed to the corresponding author.

## Ethics statement

The animal study was reviewed and approved by Ethical committee of the KU Leuven; P090/2019.

## Author contributions

LV and AV conceptualized and designed the study. LV, SD, IJ, and performed experiments and acquired data. LV, MM, and AV performed analysis and interpretation of the data. LV, SD, MM, JP, GC, and AV wrote the manuscript. All authors contributed to the article and approved the submitted version.

## Supplementary material

Supplementary Figure 1 µCT analysis of female systemic *Nrp2^-/-^* mice. Trabecular (upper panels) and cortical (lower panels) analysis of tibia of 8-week-old female *Nrp2^-/-^* mice and there wildtype littermates. All data are expressed as mean and SD. Two-tailed Student’s t-tests were performed to detect differences between *Nrp2^+/+^* and *Nrp2^-/-^* mice.

Supplementary Figure 2 Bone phenotype of osteoblast-specific *Nrp2^Ob-^* mice. (A) µCT analysis and evaluation of trabecular (upper panels) and cortical (lower panels) bone parameters of tibias of 8- and 26-week-old female mice (n=10). Two-way ANOVA analysis, with genotype and age as independent variables, followed by Sidak’s multiple comparisons test, was performed to evaluate significant differences. Representative pictures of the cortical bone of 26-week-old female *Nrp2^Ob-^* mice and their wildtype littermates are shown. (B) qPCR analysis of the osteoblast markers *Tnap*, *osteopontin* and *osteocalcin* in flushed tibia of 10-week-old male *Nrp2^Ob-^* mice and their wildtype littermates. (C) qPCR analysis of the osteoblast marker *osteocalcin* and the osteoclast marker cathepsin K in whole bone homogenates of 10-week-old male (left panel) and female (right panel) *Nrp2^Ob-^* mice and their wildtype littermates. All data are expressed as mean and SD. Two-tailed Student’s t-tests were performed to detect significant differences between *Nrp2^Ob+^* and *Nrp2^Ob-^* mice.

Supplementary Figure 3 Normal *in vitro* mineralization of calvarial osteoblasts derived from *Nrp2^Ob-^* mice. Representative alizarin red stainings of mineralizing cultures of *Nrp2^Ob+^* and *Nrp2^Ob^* ^-^ calvarial osteoblasts and qPCR analysis of the osteoblast markers *Runx2*, *Tnap*, *Osteopontin* and *Osteocalcin*. Data are expressed as mean and SEM. Two-tailed Student’s t-tests were performed to detect significant differences between *Nrp2^Ob+^* and *Nrp2^Ob-^* mice.

Supplementary Figure 4 Bone phenotype of osteoclast-specific *Nrp2^Oc-^* mice. µCT analysis and evaluation of trabecular (upper panels) and cortical (lower panels) bone parameters of tibias of 8- and 16-week-old female mice (n=8-10). All data are expressed as mean and SD. Two-way ANOVA analysis, with genotype and age as independent variables, followed by Sidak’s multiple comparisons test, was performed to evaluate significant differences.
