# Supplementary figures and images for: Neuropilin 2 in osteoblasts regulates trabecular bone mass in male mice
Source: Front Endocrinol (Lausanne). 2023 Aug 1;14:1223021. doi: 10.3389/fendo.2023.1223021 (PMC10436209; doi:10.3389/fendo.2023.1223021)

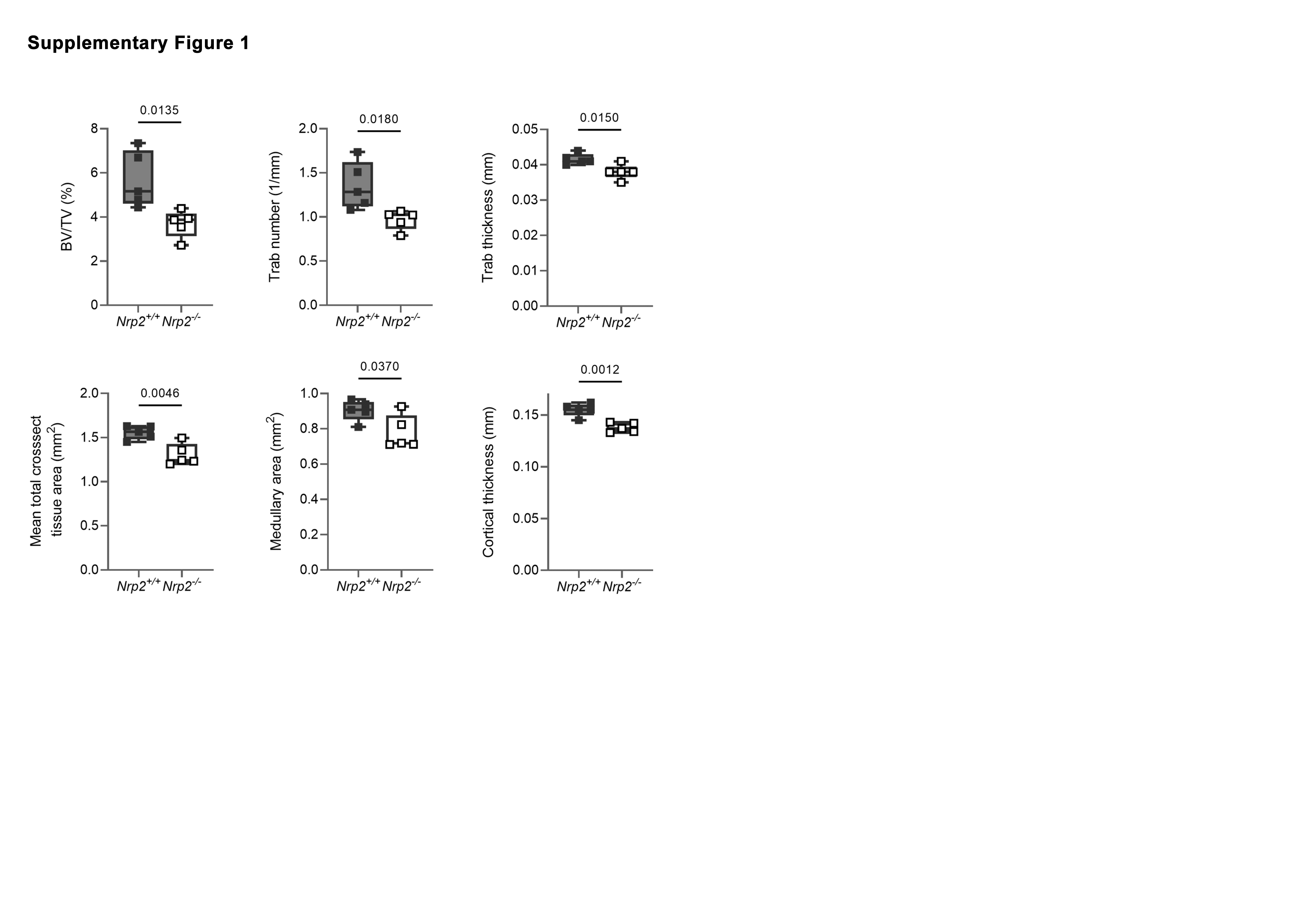

Supplement: Supplementary Figure 1 — µCT analysis of female systemic Nrp2-/- mice. Trabecular (upper panels) and cortical (lower panels) analysis of tibia of 8-week-old female Nrp2-/- mice and there wildtype littermates. All data are expressed as mean and SD. Two-tailed Student’s t-tests were performed to detect differences between Nrp2+/+ and Nrp2-/- mice. [file Image_1.tif]

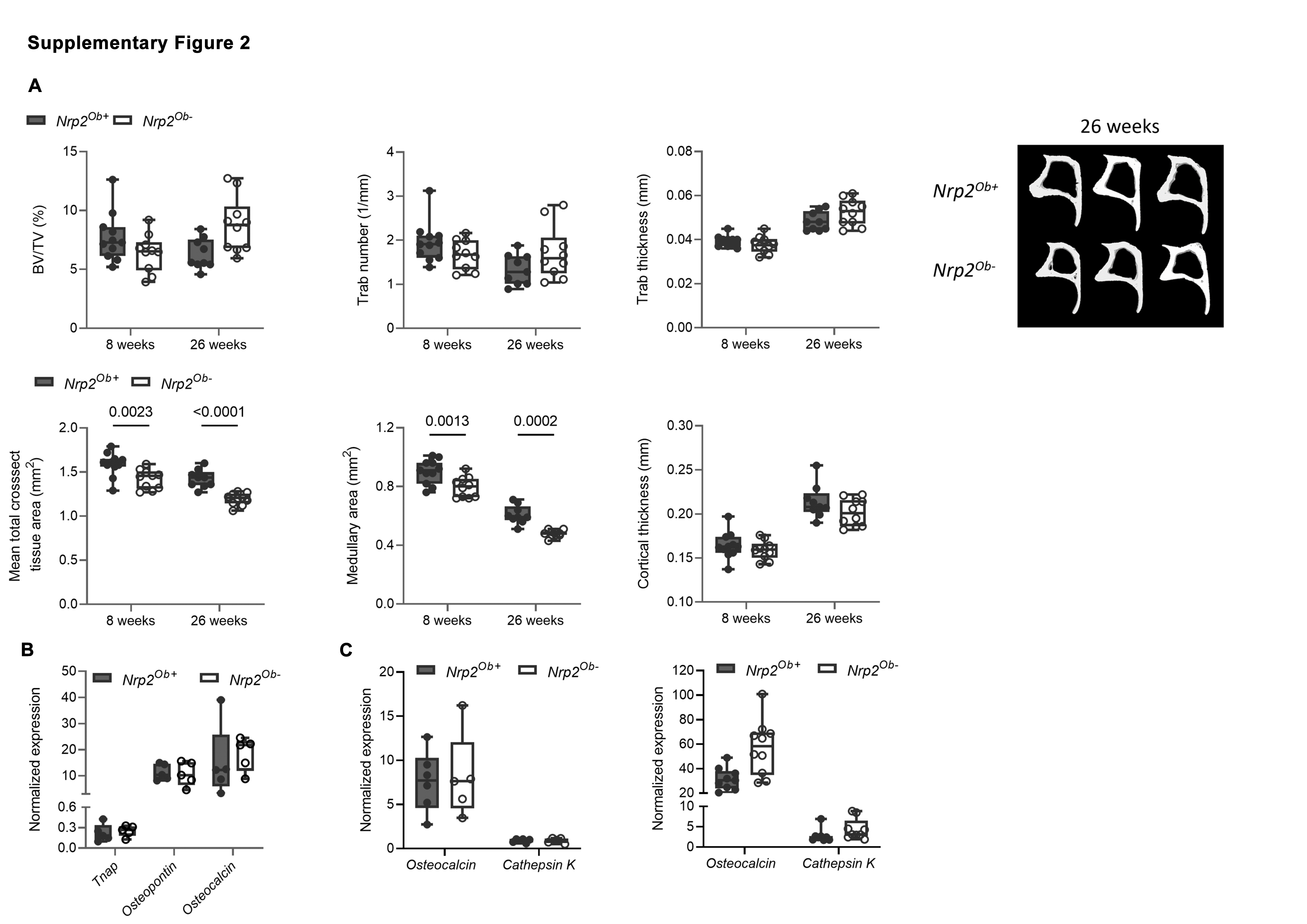

Supplement: Supplementary Figure 2 — Bone phenotype of osteoblast-specific Nrp2Ob- mice. (A) µCT analysis and evaluation of trabecular (upper panels) and cortical (lower panels) bone parameters of tibias of 8- and 26-week-old female mice (n=10). Two-way ANOVA analysis, with genotype and age as independent variables, followed by Sidak’s multiple comparisons test, was performed to evaluate significant differences. Representative pictures of the cortical bone of 26-week-old female Nrp2Ob- mice and their wildtype littermates are shown. (B) qPCR analysis of the osteoblast markers Tnap, osteopontin and osteocalcin in flushed tibia of 10-week-old male Nrp2Ob- mice and their wildtype littermates. (C) qPCR analysis of the osteoblast marker osteocalcin and the osteoclast marker cathepsin K in whole bone homogenates of 10-week-old male (left panel) and female (right panel) Nrp2Ob- mice and their wildtype littermates. All data are expressed as mean and SD. Two-tailed Student’s t-tests were performed to detect significant differences between Nrp2Ob+ and Nrp2Ob- mice. [file Image_2.tif]

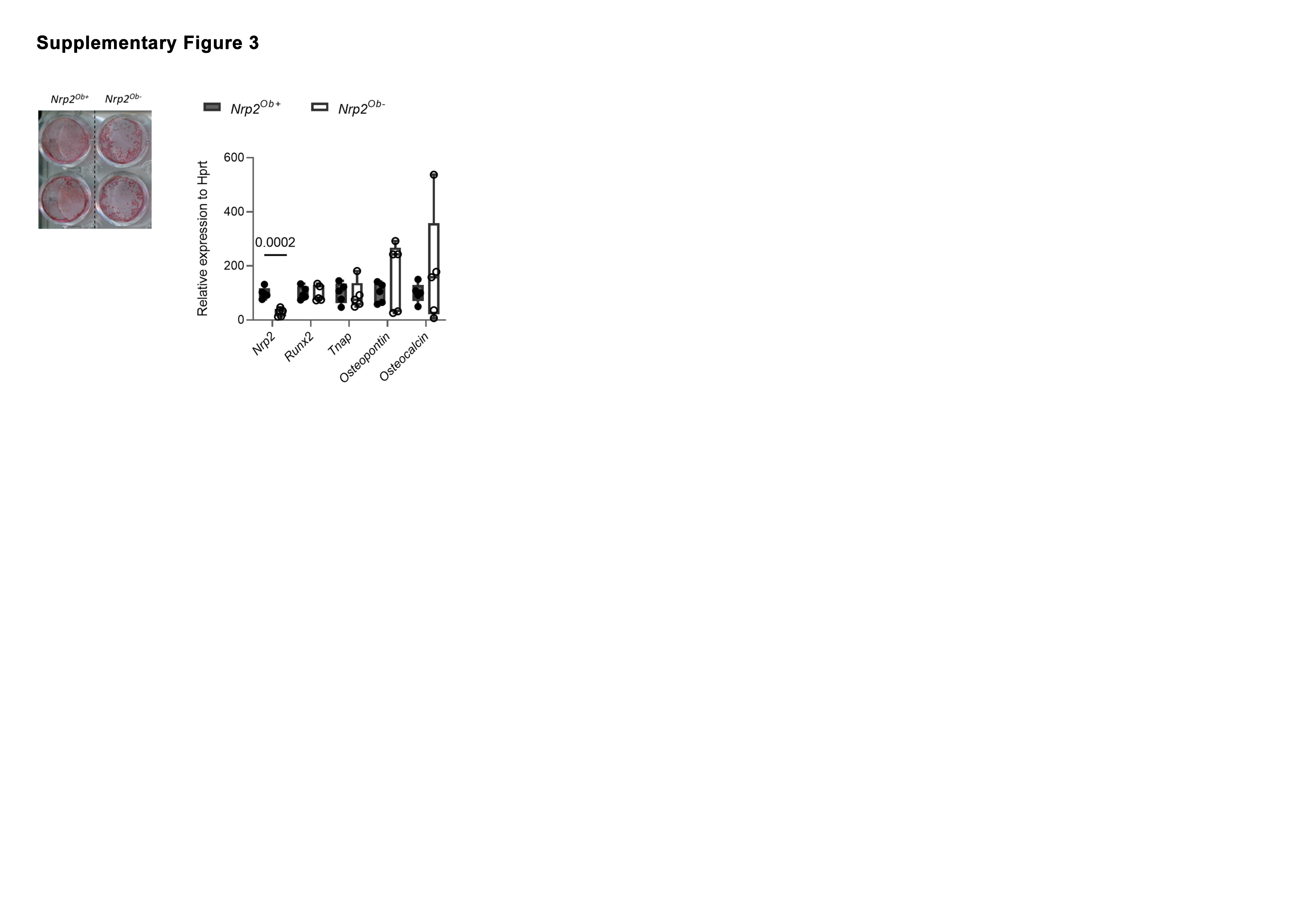

Supplement: Supplementary Figure 3 — Normal in vitro mineralization of calvarial osteoblasts derived from Nrp2Ob- mice. Representative alizarin red stainings of mineralizing cultures of Nrp2Ob+ and Nrp2Ob - calvarial osteoblasts and qPCR analysis of the osteoblast markers Runx2, Tnap, Osteopontin and Osteocalcin. Data are expressed as mean and SEM. Two-tailed Student’s t-tests were performed to detect significant differences between Nrp2Ob+ and Nrp2Ob- mice. [file Image_3.tif]

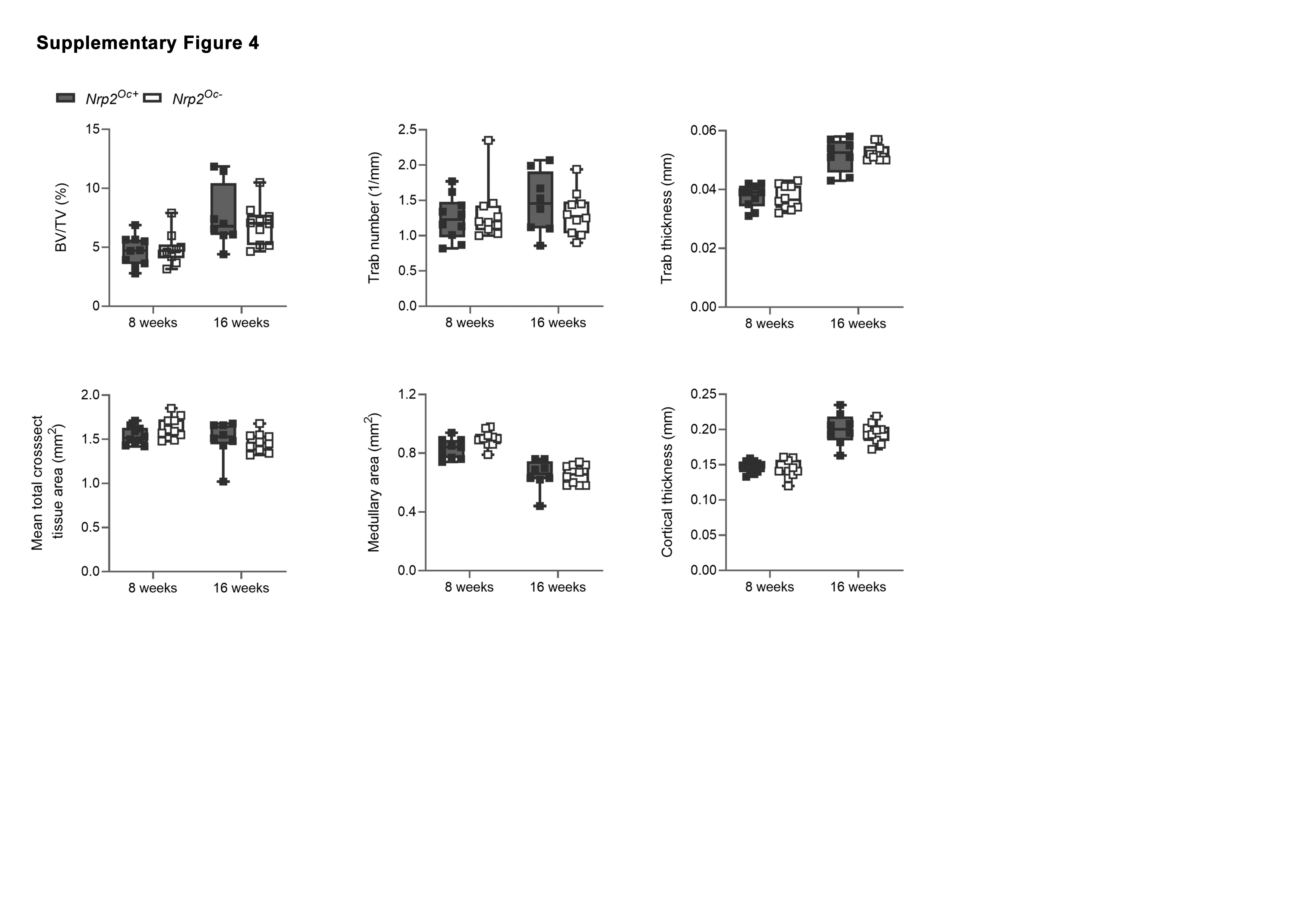

Supplement: Supplementary Figure 4 — Bone phenotype of osteoclast-specific Nrp2Oc- mice. µCT analysis and evaluation of trabecular (upper panels) and cortical (lower panels) bone parameters of tibias of 8- and 16-week-old female mice (n=8-10). All data are expressed as mean and SD. Two-way ANOVA analysis, with genotype and age as independent variables, followed by Sidak’s multiple comparisons test, was performed to evaluate significant differences. [file Image_4.tif]
